# Comprehensive Survey of miRNA-mRNA Interactions Reveals That Ccr7 and Cd247 (CD3 zeta) are Posttranscriptionally Controlled in Pancreas Infiltrating T Lymphocytes of Non-Obese Diabetic (NOD) Mice

**DOI:** 10.1371/journal.pone.0142688

**Published:** 2015-11-25

**Authors:** Thais A. Fornari, Paula B. Donate, Amanda F. Assis, Claudia Macedo, Elza T. Sakamoto-Hojo, Eduardo A. Donadi, Geraldo A. Passos

**Affiliations:** 1 Molecular Immunogenetics Group, Department of Genetics, Ribeirão Preto Medical School, University of São Paulo (USP), 14049–900, Ribeirão Preto, SP, Brazil; 2 Department of Biology, Faculty of Philosophy, Sciences and Letters of Ribeirão Preto, USP, 14040–900, Ribeirão Preto, SP, Brazil; 3 Department of Medicine, Division of Clinical Immunology, Ribeirão Preto Medical School, USP, 14049–900, Ribeirão Preto, SP, Brazil; 4 Department of Morphology, Physiology and Basic Pathology, Disciplines of Genetics and Molecular Biology, School of Dentistry of Ribeirão Preto, USP, 14040–904, Ribeirão Preto, SP, Brazil; University of Siena, ITALY

## Abstract

In autoimmune type 1 diabetes mellitus (T1D), auto-reactive clones of CD4^+^ and CD8^+^ T lymphocytes in the periphery evolve into pancreas-infiltrating T lymphocytes (PILs), which destroy insulin-producing beta-cells through inflammatory insulitis. Previously, we demonstrated that, during the development of T1D in non-obese diabetic (NOD) mice, a set of immune/inflammatory reactivity genes were differentially expressed in T lymphocytes. However, the posttranscriptional control involving miRNA interactions that occur during the evolution of thymocytes into PILs remains unknown. In this study, we postulated that miRNAs are differentially expressed during this period and that these miRNAs can interact with mRNAs involved in auto-reactivity during the progression of insulitis. To test this hypothesis, we used NOD mice to perform, for the first time, a comprehensive survey of miRNA and mRNA expression as thymocytes mature into peripheral CD3^+^ T lymphocytes and, subsequently, into PILs. Reconstruction of miRNA-mRNA interaction networks for target prediction revealed the participation of a large set of miRNAs that regulate mRNA targets related to apoptosis, cell adhesion, cellular regulation, cellular component organization, cellular processes, development and the immune system, among others. The interactions between miR-202-3p and the Ccr7 chemokine receptor mRNA or Cd247 (Cd3 zeta chain) mRNA found in PILs are highlighted because these interactions can contribute to a better understanding of how the lack of immune homeostasis and the emergence of autoimmunity (e.g., T1D) can be associated with the decreased activity of Ccr7 or Cd247, as previously observed in NOD mice. We demonstrate that these mRNAs are controlled at the posttranscriptional level in PILs.

## Introduction

Faulty negative selection in the thymus allows for the emigration of autoreactive T lymphocytes to the periphery, which can trigger pathogenic autoimmunity [1–5].

An example of a disease resulting from defective negative selection, although environmental factors also compete, is type 1 diabetes mellitus (T1D), which is a prototype of autoimmune disease [6]. In this disease, self-reactive CD4^+^ and CD8^+^ T lymphocytes, which mainly recognize insulin and/or other pancreatic autoantigens (e.g., GAD 65), infiltrate the pancreas (here, these cells are termed pancreas-infiltrating T lymphocytes or PILs) and destroy the insulin producing beta-cells through inflammatory insulitis. This is a gradual process that takes several years in prone individuals or over five to eight months in nonobese diabetic (NOD) mice [7–12]. Specific autoantibodies also participate in the destruction of beta cells in T1D [13].

As we introduced in a previous work [14], the NOD mouse is an autoimmune mouse strain that represents the most appropriate animal model to immune tolerance and develop autoimmune T1D, which reflects at least a part of human T1D [15–19].

Susceptibility to T1D is often associated with the major histocompatibility humans and the mouse homolog I-Ag in the NOD mouse [20–24], although non-MHC genes, such as Ptpn22, also participate in susceptibility to this disease [25]. Moreover, the NOD mouse features insulin-dependent (*Idd*) susceptibility loci. However, the contribution of various other factors to the immunopathogenesis of T1D remains largely unknown [19,21,26].

Type 1 diabetes mellitus does not occur without the participation of CD3^+^ T lymphocytes with either a CD4^+^ or CD8^+^ phenotype [27,28]. The immunoregulatory genes or cells that control the aggressiveness of autoreactive in the periphery and peripheral tolerance is a matter of importance that was first recognized several years ago and has been the focus of attempts to better understand the molecular genetic basis of autoimmunity in T1D [29–39].

We previously demonstrated that the development of T1D in NOD mice follows the transcriptional levels of immune reactivity genes during the maturation of thymocytes to peripheral CD3^+^ T lymphocytes [14]. However, the fine control of this gene expression, e.g., posttranscriptional control involving microRNAs (miRNAs), that occurs in these cells, including PILs, is largely unknown. MiRNAs regulate the gene expression of a variety of biological processes at the posttranscriptional level and are considered to be the main fine-tuning controllers of normal and pathological development [40–42].

Taking into account the function of miRNAs as posttranscriptional regulators, in this work, we sought to identify the miRNAs that are differentially expressed as well as their mRNA targets by comparing samples of thymocytes, peripheral CD3^+^ T lymphocytes and PILs during the progression of insulitis in NOD mice.

To answer these questions, we comprehensively assayed, for the first time and in a comparative manner, the mirnome (miRNAs) and transcriptome (mRNAs) of these cells using microarray hybridizations.

The data were first analyzed by hierarchical clustering to identify the differentially expressed miRNAs and mRNAs, and a selected set of these RNA species were reanalyzed using a dedicated bioinformatics tool (GenMir++ algorithm) to evaluate their interaction networking.

From the set of miRNA-mRNA interactions that were found during the development of thymocytes into PILs, we identified interactions between miR- 202-3p and the Ccr7 chemokine receptor and Cd247 (Cd3 zeta chain) mRNAs, which were previously found to be involved in the control of aggressive autoimmunity in T1D in NOD mice.

## Materials and Methods

### Animals and assessment of type 1 diabetes mellitus

Female non-obese diabetic (NOD) mice were born in specific pathogen free (SPF) conditions at the special mouse facility of the Ribeirão Preto Medical School and maintained in our laboratory in SPF mini-isolators with sterilized water and food *ad libitum*. The pre-diabetic mice were 1 month ± 2 week-old (1 mo) or 7 month ± 4 week-old (7 mo) mice. The 7 mo animals that became diabetic, have been identified and included in the study (group of diabetic 7 mo mice). Diabetes was assessed based on the blood glucose levels (animals exhibiting ≥ 250 mg glucose/dl blood were considered diabetic) using Accu Check-Active (Roche Diagnostics Brazil, São Paulo, Brazil). All experiments in this study were made in triplicate (samples collected from three animals of each phase of the disease) for microarray hybridizations as well as for qRT-PCRs.

The protocols used in this study were approved by the local ethics committee on animal experimentation (CEUA, University of São Paulo at Ribeirão Preto Campus, Brazil, Permit No. 120/2008).

### Assessment of insulitis

The progression of insulitis was assessed by conventional hematoxylin and eosin (H&E) histology of the pancreas from pre-diabetic or diabetic NOD mice. The tissues were included in paraffin blocks, sliced at a 5-μm thickness, mounted on slides and stained with H&E. Histological examination and capturing of images were performed with a Leica model DMLB2 microscope coupled to a Leica DC300F camera. This approach allowed the observation of pancreatic islets with or without lymphocyte infiltration (data not shown).

### Isolation of thymocytes, peripheral CD3^+^ T cells and PILs

The thymocytes or peripheral CD3^+^ T cells were isolated from the thymus or spleen, respectively, of pre-diabetic or diabetic NOD mice according to a previously described protocol [43]. In summary, thymi were dissected in DMEM/F10 medium, thymocytes were obtained by 2–3 passages of the thymic fragments through a 10-μm mesh nylon membrane (Sefar Inc., Depew, NY, USA), and pelleted thymocytes were suspended in PBS. Fluorescence activated cell sorting (FACS) analysis using a BD-FACSCalibur flow cytometer with a phycoerythrin (PE)-labeled anti-CD3 antibody indicated that this procedure yielded a thymocyte population with purity of ≥ 93%. These cells were used for total RNA extraction.

The peripheral CD3^+^ T cells were isolated from spleens of mice using magnetic beads for negative selection (Pan T-cell isolation kit, mouse, Miltenyi Biotec) according to the manufacturer´s instructions. FACS analysis with the PE-labeled anti-CD3 antibody indicated that this procedure yielded a peripheral CD3^+^ T lymphocyte population of approximately 87% purity. These cells were used for total RNA extraction.

The T CD3^+^ pancreas-infiltrating lymphocytes (PILs) were isolated from 4 week-old pre-diabetic animals. Pancreata from pre-diabetic NOD female mice were dissected and macerated in RPMI 1640 medium. The pancreatic fragments were then incubated in 5 ml of 0.125% (w/v) type II collagenase and 0.1 DNase I in RPMI 1640 medium at 37°C for 15 minutes and gently agitated every 5 minutes with a 1 ml pipette. After digestion, the T lymphocytes were separated by 2–3 passages of the fragments and culture medium throughout a 10 um mesh nylon membrane (Sefar Inc, Depew, NY, USA). For T CD3^+^ cell recovery, the filtrate was centrifuged for 10 minutes at 1300 rpm at 4°C. Pelleted cells were suspended in RPMI 1640 medium and stained with a FITC-labeled anti-mouse CD3e antibody (Biolegend) for 30 minutes at 4°C. After that, T CD3^+^ cell population was separated by fluorescent-activated cell sorting in a FACS Aria III (BD Biosciences) flow cytometer. This procedure yielded approximately 70.8% purity of PIL T CD3^+^ cells (S1 Fig). These cells were used for total RNA extraction.

### Total RNA extraction and quality control

The total RNA was extracted from approximately 1 x 10^7^ thymocytes, peripheral CD3^+^ T cells or PILs using the mirVana total RNA isolation kit (Ambion, NY, USA) according to the manufacturer´s instructions. The parameters for the quality control of the RNA preparations were previously described [43]. RNA preparations were confirmed to be free of proteins and phenol by UV spectrophotometry.

The integrity of RNA species was assessed by microfluidic electrophoresis using Agilent 6000 RNA Nanochips and an Agilent 2100 Bioanalyzer (Agilent Technologies, Santa Clara, CA, USA). Only RNA samples that were free of proteins and phenol and featured an RNA Integrity Number (RIN) ≥ 9.0 were used (data not shown).

### Microarray hybridizations

#### For mirnome (miRNA) profiling

We used total RNA for direct labeling with Cy3 using the Agilent miRNA Complete Labeling and Hybridization Kit (Agilent Technologies, Mississauga, ON, Canada). The Cy3-labeled RNA samples were hybridized to Agilent mouse 8 x 15 K-format oligonucleotide miRNA microarrays, and the slides were washed according to the manufacturer´s instructions (Agilent Technologies) and scanned using an Agilent DNA microarray scanner [43].

#### For transcriptome (mRNA) profiling

We used total RNA to synthesize dscDNA and cyanine 3 (Cy3)-CTP-labeled complementary amplified RNA (cRNA) using the Agilent Linear Amplification Kit (Agilent Technologies, Santa Clara, CA, USA) according to the manufacturer´s instructions. The (Cy3)-cRNA samples were hybridized to Agilent mouse 4 x 44 K oligonucleotide microarrays (Agilent Technologies) for 18 h at 60°C, washed according the manufacturer´s instructions and scanned using an Agilent DNA microarray scanner [43].

### Microarray data analysis

**Fig 1:**
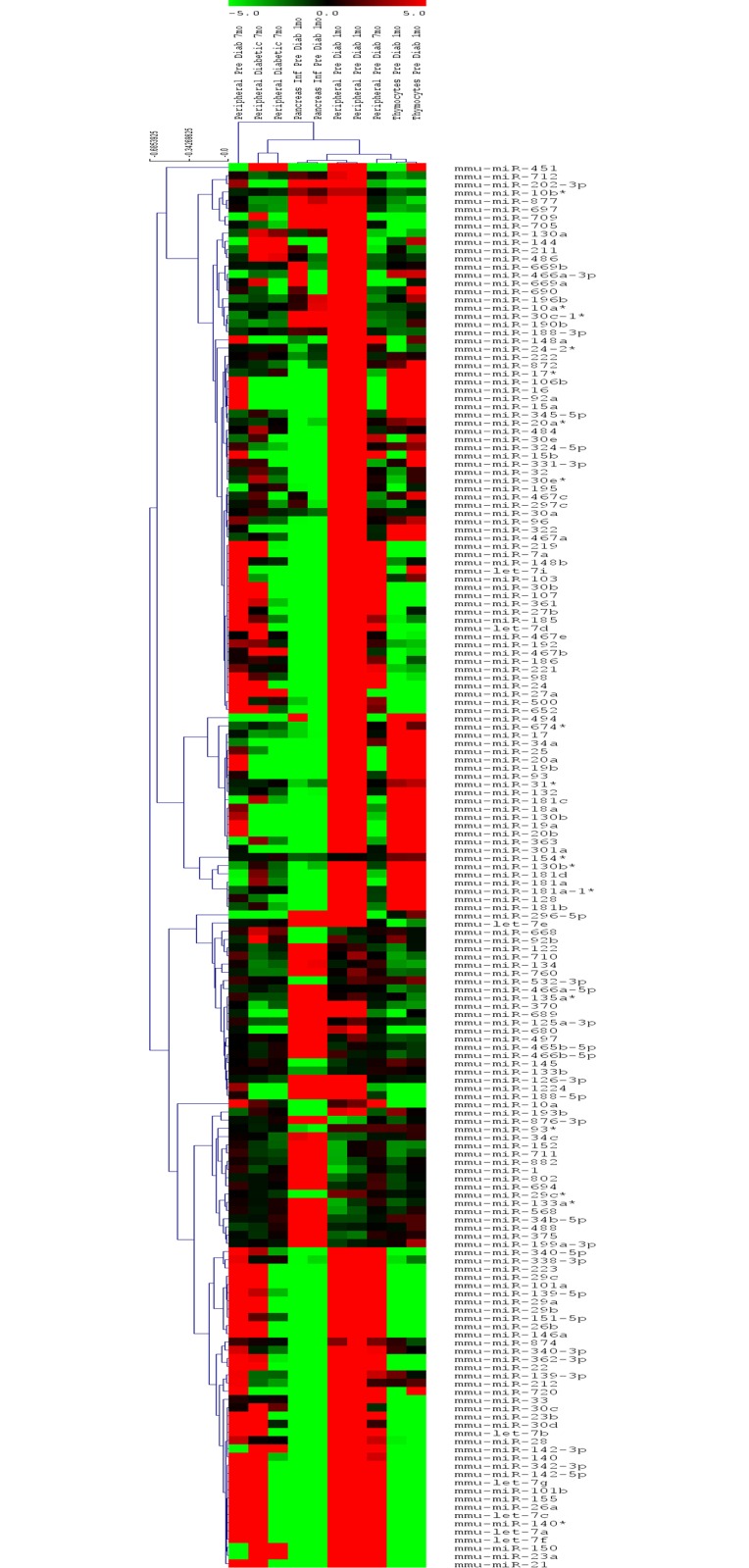
Mirnome (miRNA) expression profiles. The samples analyzed were as follow: Thymocytes (n = 2) or pancreas-infiltrating lymphocytes (PILs) (n = 2) derived from pre-diabetic (1 mo) or peripheral CD3^+^ T lymphocytes derived from pre-diabetic (1 mo n = 2 or 7 mo n = 3) NOD mice. These cell types featured unique mirnome expression signature. The dendrograms and heat map were obtained using cluster and tree-view algorithms through the Agilent GeneSpring platform. Heat map legend: red = up-regulation, green = down-regulation, black = unmodulated (Pearson correlation metrics).

The hybridization signals from the scanned miRNA or mRNA oligonucleotide microarrays were extracted using the Agilent Feature Extraction software, version 10.5. The expression profiles from independent preparations of thymocytes or PILs from pre-diabetic or peripheral CD3^+^ lymphocytes from pre-diabetic or diabetic animals were analyzed by comparing the microarray hybridizations of the respective samples (Fig 1). The numerical, quantitative microarray data were normalized to the 75th percentile and analyzed using the GeneSpring GX bioinformatics platform (http://www.agilent.com/chem/genespring) according to the default instructions and allowing for hierarchical clustering of samples of mice or types of RNAs through ANOVA statistical analysis (P < 0.05), with a fold change ≥ 2.0 and uncentered Pearson correlation metrics [44].

Dendrograms were used to represent the similarities and dissimilarities in expression profiles between samples, miRNAs or mRNAs, and a colored heat map was used to represent the variability of RNA expression. The GeneSpring platform or Panther gene classification system (http://www.pantherdb.org/) was used to assess the biological functions of mRNAs. Information about the miRNAs was retrieved from the miRBase data bank (www.mirbase.org).

The microarray data of this study are available online at EMBL EBI ArrayExpress Data Bank (https://www.ebi.ac.uk/arrayexpress/) under Array Express accessions E-MEXP-3572 (miRNAs of thymocytes and peripheral T CD3^+^ lymphocytes), E-MEXP-3573 (mRNAs of thymocytes and peripheral T CD3^+^ lymphocytes), E-MTAB-3556 (miRNAs of PILs) and E-MTAB-3555 (mRNAs of PILs).

### Quantitative reverse-transcription real-time PCR (qRT-PCR)

#### Cd247 mRNA

The differential expression of Cd247 mRNA as observed by microarray hybridizations was confirmed using qRT-qPCR. Complementary DNA (cDNA) was synthesized from 500 ng of total RNA using High-Capacity RNA-to-cDNA Kit (Applied Biosystems—Life Technologies, USA) as recommended. The PCR products were amplified from cDNA samples using Applied Biosystems TaqMan Universal PCR Master Mix (Applied Biosystems—Life Technologies, USA) with an assay probe spanning the exon junction of the murine Cd247 (FAM/MGB probe; assay ID: Mn00446171_m1 from Applied Biosystems—Life Technology, USA) or for the Applied Biosystems mouse GAPDH endogenous control (FAM/MGB Probe, Non-Primer Limited; assay ID: Mm00446171_m1) that was used as constitutive reference.

#### miR-202-3p

For qRT-PCR confirmation of miRNA levels we used TaqMan MicroRNA Assay (Applied Biosystems—Life Technologies, USA) to evaluate the expression of miR-202-3p (accession number: MIMAT0000235; mature sequence: AGAGGUAUAGCGCAUGGGAAGA). The snoRNA202 (accession number: AF357327; sequence: GCTGTACTGACTTGATGAAAGTACTTTTGAACCCTTTTCCATCTGATG) was used as an endogenous control. This method uses two-step qRT-PCR. In the reverse transcription (RT) step, cDNA was reverse transcribed from 10 ng of total RNA samples using the miRNA-specific stem-loop primers (sequences above) from the TaqMan MicroRNA Assays and reagents from the TaqMan MicroRNA Reverse Transcription kit (Applied Biosystems—Life Technologies, USA). PCR products were amplified from each cDNA sample using the TaqMan MicroRNA Assays (Applied Biosystems—Life Technologies, USA), which contains the specific probes labeled with FAM, together with the TaqMan Universal PCR Master Mix (Applied Biosystems—Life Technologies, USA), according to manufacturer's instructions.

Transcriptional expression levels were determined using a StepOne Real-Time PCR System (Applied Biosystems, USA). The ΔΔCT was used as a relative normalization method. We used the GraphPad Prism 5.00 tool (http://www.graphpad.com/prism/Prism.html) to run one-way or two-way ANOVA with Bonferroni’s correction statistics.

### Reconstruction of miRNA-mRNA interaction networks

We used a protocol that was previously designed by our group and explained in detail [43]. Briefly, the miRNA-mRNA interaction networks were reconstructed on the basis of the respective miRNA and mRNA expression profiles to identify candidate miRNA-mRNA target pairs that were best supported by the expression data. The GenMir++ algorithm [45,46], which is available at http://www.psi.toronto.edu/genmir/, was used to establish such interactions. This algorithm calculates the scores to reproduce a mRNA profile using a weighted combination of the genome-wide, average normalized expression profile and the negatively weighted profiles of a subset of the miRNA regulators. The networks were graphically represented using the Cytoscape version 2.1 program (www.cytoscape.org). We used the TargetScanS database (http://www.targetscan.org) to verify the prediction of the selected target mRNAs.

### Validations of miRNA-mRNA interactions

#### Determination of the hybridization minimum free energy (mfe)

Using the miRNA-mRNA interaction networks generated by the GenMir++ algorithm, we selected pairs based on the biological function of the mRNA target. The annealing was validated through the use of the RNA-hybrid algorithm [47,48], which estimates the most favorable pairing between a given miRNA and its mRNA target by calculating the minimum free energy based on a thermodynamic state that postulates that an RNA duplex is more stable and thermodynamically stronger when the free energy is low [43,49]. This program is also available at http://bibiserv.techfak.uni-bielefeld.de/rnahybrid/.

#### Luciferase reporter gene assay (LRGA)

We used a protocol previously designed in our group and explained in detail [43]. Briefly, the complementary oligonucleotide pairs containing a portion of the predicted miRNA-binding sites of the Ccr7 or Cd247 (CD3 zeta) 3´ UTRs were synthesized by Integrated DNA Technologies (IDT, Coralville, IA, USA). The oligonucleotides were annealed and cloned into the pmirGLO vector (Promega Corporation, USA) between the XhoI/XbaI restriction sites of the polycloning site (PCS), resulting in the miRNA target region in the correct 5´ to 3´ orientation and immediately downstream of the luciferase gene. For selected targets, we introduced point mutations into the 7-nt seed-binding sequence. These constructs, named "pMIR-Ccr7” or “pMIRCd247” for the wild-type sequences and "pMIR-Ccr7(m)” or “pMIR-Cd247(m)” for the mutant sequences, were selected by colony-polymerase chain reaction (PCR) using a pair of primers flanking the vector PCS. We used *Escherichia coli* DH5α for cloning.

For the LRGA, 0.2 μg of each pmirGLO construct was transfected with 1.6 pmol of miR-202-3p or scrambled control miRNA (Thermo Scientific Dharmacon, Waltham, MA, USA) into human HEK-293T cells (6 x 10^4^ cells/well) in a 96-well plate. Transfections were performed using Attractene Transfection Reagent (Qiagen, Hilden, Germany) according to the manufacturer´s instructions. Transfected cells were incubated at 37°C in a 5% CO2 incubator, and 24 h after transfection, the cells were lysed in Passive Lysis Buffer. Firefly and renilla luciferase activities were measured in a Molecular Devices FlexStation 3 Luminometer using the Dual-Luciferase reporter system (Promega Corporation, USA) according to the manufacturer´s instructions. Our laboratory has national biosafety permission (National Technical Committee for Biosafety, Brasilia, Brazil, CTNBio, Permit No. 0040/98).

### Statistical analysis

The LRA results are presented as the standard error of the mean (SEM). The differences were evaluated by one-way ANOVA followed by Student´s ttest (two groups). P < 0.05 was considered statistically significant.

## Results

### Transcriptional profiles in thymocytes, peripheral T CD3^+^ lymphocytes and PILs

#### Microarray miRNA expression profiles (mirnome)

From the set of murine miRNAs that were described and present in the microarray, 171 were identified as differentially expressed [p < 0.05, false discovery rate (FDR) = 0.05 and fold change ≥ 2.0] when we compared thymocytes from 1 mo-old and CD3^+^ peripheral T lymphocytes from 1 mo-old or 7 mo-old pre-diabetic mice with the corresponding cells from ≥ 7 mo-old diabetic mice or PILs from pre-diabetic 1mo-old NOD mice. The dendrogram and heat map depicted in Fig 1 show that these cell types feature unique miRNA expression signatures. From this set of miRNAs, we then selected 26 whose respective expression was exclusive in a given cell type of pre-diabetic or diabetic animals, i.e., induced/repressed in thymocytes, CD3^+^ peripheral T lymphocytes or PILs (Table 1). The up- or down-regulated miRNAs from each cell type were then reanalyzed using the GenMir++ algorithm.

**Table 1:** Modulated miRNAs according to cell type during the transition from pre-diabetic to diabetic NOD mice, their potential mRNA targets as predicted by the GenMir++ algorithm, the number of miRNA-mRNA interactions and GO biological processes of predicted target mRNAs. (↓) Down-regutated, (↑) Up-regulated.

Cell type	Selected miRNAs	mRNA targets	GO Biological processes
Thymocytes (269 interactions)	miR-101a (↓)	Bub1 Bub1b Cenpf Endou Rorc Pax1 Epha2 Rag1 Ptcra	Biological regulation (GO: 0065007), Cellular (GO:0009987) Developmental (GO: 0032502), Immune system (GO:0002376), Metabolic (GO: 0008152), Multicellular organismal (GO: 0032501), Response to stimulus (GO: 0050896)
	miR-29c (↓)	Aatk Adamtsl4 Bcl2l14 Cd5l Chac1 Clu Lpar1 Naip2 Rhob Agtr1a Cd38 Ahsp Alox12 Ank1 Bcl11a Hhex Ccl3 Ccl6 Cd36 Csf1r Itgam Lilrb3 Cd55 Cebpb Hoxb7 Klf1 Tbx21 Chst4 Clec4d Cplx2 Crkl Csf3r Spon2 Cxcr5 Daf2 Elf4 Eomes Hmox Lmo2 Enpp1 Fcgrt Fgfr1 Hdac9 Hmox1 Igj Il1b Irak3 Nod1 Pf4 Psen2 Sgk1 Smap1 Sphk1 Spna1 Tgm2 Tlr4 Trim10	Apoptotic (GO:0006915), Adhesion (GO:0022610), Regulation (GO:0065007), Cellular component organization (GO:0071840), Cellular (GO:0009987), Developmental (GO:0032502), Immune system (GO:0002376), Localization (GO:0051179), Metabolic (GO:0008152), Multicellular organismal (GO:0032501), Response to stimulus (GO:0050896), Reproduction (GO:0000003)
	miR-345-5p (↑)	2010001M09Rik Aatk Adamtsl4 Bcl2l14 Casp1 Cd5l Fasl Hdac9 Hmox1 Agtr1a (angiotensin receptor) Ahsp Aldh1a1 Alox12 (leukotriene synthase activity) Ambra1 Ank1 App Anxa1 Bcl11a Ccl3 Ccl6 Cd36 Cd38 Cd55 Cd74 Cebpb Elf4 Eomes Chac1 Chst4 Clcf1 Csf1 (cytokine) Csf1r Csf3r Cxcr5 Clec4a2 Clec4d Clu Dapl1 Col18a1 Cplx2 Crkl Daf2 Enpp1 Fastkd3 Fcgrt Fgfr1 Fgl2 Fosl1 Fyb Hhex Hoxb7 Hpx Hspa1b Igj Il18r1 Il1b Irak3 Itgam Kcnip3 Klf1 Klre1 Lbp Lilrb3 Lmo2 Lpar1 Meis1 Naip2 Naip5 Niacr1 Nod1 Nrbp2 Pf4 Pglyrp1 Polr3c Prop1 Psen2 Rhob Samhd1 Sgk1 Slc11a1 Smap1 Snca Sphk1 Spna1 Spon2 Tbx21 Tcf15 Tctn3 Terc Tgm2 Tlr4 Trim10 Unc93b1 Zbtb32	Apoptotic (GO:0006915), Adhesion (GO:0022610), Regulation (GO:0065007), Cellular component organization (GO:0071840), Cellular (GO:0009987), Developmental (GO:0032502), Immune system (GO:0002376), Localization (GO:0051179), Metabolic (GO:0008152), Multicellular organismal (GO:0032501), Response to stimulus (GO:0050896), Reproduction (GO:0000003)
	miR-34a (↑)	2010001M09Rik Aatk Adamtsl4 Agtr1a Ahsp Aldh1a1 Alox12 Ambra1 Ank1 Anxa1 App Bcl11a Bcl2l14 Casp1 Ccl3 Ccl6 Cd300lf Cd36 Cd38 Cd55 Cd5l Cd74 Cd79a Cebpb Chac1 Chia Chst4 Clcf1 Clec4a2 Clec4d Clu Col18a1 Cplx2 Crkl Csf1 Csf1r Csf3r Cxcr5 Daf2 Dapk2 Dapl1 Dnase1l3 Elf4 Enpp1 Eomes Epas1 Fasl Fastkd3 Fcgr3 Fcgrt Fgfr1 Fgl2 Fosl1 Fyb H2-Q10 Hdac9 Herpud1 Hhex Hipk2 Hmox1 Hoxb7 Hpx Hspa1b Igj Il18r1 Il1b Il1rl1 Irak3 Itgam Kcnip3 Klf1 Klre1 Lbp Lilrb3 Lmo2 Lpar1 Map3k9 Masp2 Meis1 Myo1e Naip2 Naip5 Niacr1 Nod1 Nrbp2 Pf4 Pglyrp1 Polr3c Prop1 Psen2 Rhob Samhd1 Sgk1 Slc11a1 Smap1 Snca Sphk1 Spna1 Spon2 Tbx21 Tcf15 Tctn3 Terc Tgm2 Tlr4 Trim10 Unc93b1 Zbtb32	Apoptotic (GO:0006915), Adhesion (GO:0022610), Regulation (GO:0065007), Cellular component organization (GO:0071840), Cellular (GO:0009987), Developmental (GO:0032502), Immune system (GO:0002376), Localization (GO:0051179), Metabolic (GO:0008152), Multicellular organismal (GO:0032501), Response to stimulus (GO:0050896), Reproduction (GO:0000003)
Peripheral T Lymphocytes (58 interactions)	miR-296-5p (↓)	Bcl2l1 Bex2 Cdkn1a Col18a1 Cxcl12 Ghr Krt18 Krt8 Lpar1 Mapt Ntn1 P2rx1 Ptprf Smo Spon2 Uaca Vegfa	Apoptotic (GO:0006915), Adhesion (GO:0022610), Regulation (GO:0065007), Cellular component organization (GO:0071840), Cellular (GO:0009987), Developmental (GO:0032502), Immune system (GO:0002376), Localization (GO:0051179), Metabolic (GO:0008152), Multicellular organismal (GO:0032501), Response to stimulus (GO:0050896), Reproduction (GO:0000003)
	miR-378 (↑)	Acvr1c Bcl2l1 Bex2 Bmp7 Cckbr Ccl8 Cdkn1a Cfb Col18a1 Cxcl12 Fgfr1 Ghr Gja1 Gli3 Hba-a1 Hbb-b1 Id1 Jun Krt18 Krt8 Lpar1 Lrrc17 Mapt Mmp2 Ngf Nol3 Ntn1 P2rx1 Phlda1 Ptprf Rorc Rtkn Smo Sox4 Sox9 Spon2 Sulf1 Tgfb2 Uaca Vegfa Vtn	Apoptotic (GO:0006915), Adhesion (GO:0022610), Regulation (GO:0065007), Cellular component organization (GO:0071840), Cellular (GO:0009987), Developmental (GO:0032502), Immune system (GO:0002376), Localization (GO:0051179), Metabolic (GO:0008152), Multicellular organismal (GO:0032501), Response to stimulus (GO:0050896), Reproduction (GO:0000003)
Pancreas Infiltranting Lymphocytes -PILs (1042 interactions)	miR-20b (↓)	5830411N06Rik Amigo2 Bcl11b Btla Card11 Ccr7 Cd2 Cd247 Cd27 Cd28 Cd3d Cd3e Cd5 Cd8a Coro1a Dock2 Ets1 Gzma Il16 Il2rg Il7r Itgal Jarid2 Klhl6 Lat Lax1 Lck Madd Nlrc3 Pdcd1 Pglyrp2 Prf1 Prkcq Ptprc Rassf5 Rhoh Sash3 Satb1 Sh2d1a Sh3kbp1 Spn Stk4 Tcf7 Trat1 Vav1	Apoptotic (GO:0006915), Adhesion (GO:0022610), Regulation (GO:0065007), Cellular component organization (GO:0071840), Cellular (GO:0009987), Developmental (GO:0032502), Immune system (GO:0002376), Localization (GO:0051179), Metabolic (GO:0008152), Multicellular organismal (GO:0032501), Response to stimulus (GO:0050896), Reproduction (GO:0000003)
	miR-30b (↓)	5830411N06Rik Amigo2 Bcl11b Btla Card11 Ccr7 Cd2 Cd247 Cd27 Cd28 Cd3d Cd3e Cd4 Cd5 Cd8a Coro1a Dock2 Ets1 Gzma Hsh2d Il16 Il2rg Il7r Itgal Jarid2 Klhl6 Lat Lax1 Lck Madd Nlrc3 Pdcd1 Pglyrp2 Pik3cd Prf1 Prkcq Ptprc Rassf5 Rhoh Sash3 Satb1 Sh2d1a Sh3kbp1 Spn Stk4 Tcf7 Tnfrsf18 Trat1 Vav1 Zap70	Apoptotic (GO:0006915), Adhesion (GO:0022610), Regulation (GO:0065007), Cellular component organization (GO:0071840), Cellular (GO:0009987), Developmental (GO:0032502), Immune system (GO:0002376), Localization (GO:0051179), Metabolic (GO:0008152), Multicellular organismal (GO:0032501), Response to stimulus (GO:0050896), Reproduction (GO:0000003)
	miR-30c (↓)	5830411N06Rik Amigo2 Bcl11b Btla Card11 Ccr7 Cd1d2 Cd2 Cd247 Cd27 Cd28 Cd3d Cd3e Cd4 Cd5 Cd8a Cnr2 Coro1a Csk Dapl1 Dock2 Elmo1 Ets1 Fyb Gpr65 Gzma H2-Oa H2-Q10 Hsh2d Icam1 Ikzf1 Il16 Il27ra Il2rg Il7r Irf1 Itgal Jarid2 Klhl6 Lat Lax1 Lck Lrdd Ltb Madd Mgea5Myst3Nlrc3 Pdcd1 Pglyrp2 Pik3cd Plcg1 Prf1 Prkcq Psmb8 Ptprc Ptprv Rassf5 Rhoh Ripk3 Sash3 Satb1 Sh2d1a Sh3kbp1 Slamf7 Slc2a3 Spn Stap1 Stat1 Stk4 Tcf7 Themis Thy1 Tnfrsf18 Trat1 Ubash3a Vav1 Zap70	Apoptotic (GO:0006915), Adhesion (GO:0022610), Regulation (GO:0065007), Cellular component organization (GO:0071840), Cellular (GO:0009987), Developmental (GO:0032502), Immune system (GO:0002376), Localization (GO:0051179), Metabolic (GO:0008152), Multicellular organismal (GO:0032501), Response to stimulus (GO:0050896), Reproduction (GO:0000003)
	miR-30d (↓)	5830411N06Rik Amigo2 Bcl11b Btla Card11 Ccr7 Cd1d2 Cd2 Cd247 Cd27 Cd28 Cd3d Cd3e Cd4 Cd5 Cd8a Cnr2 Coro1a Csk Dapl1 Dock2 Elmo1 Ets1 Fyb Gzma H2-Oa H2-Q10 Hsh2d Icam1 Ikzf1 Il16 Il27ra Il2rg Il7r Irf1 Itgal Jarid2 Klhl6 Lat Lax1 Lck Lrdd Madd Mgea5 Myst3 Nlrc3 Pdcd1 Pglyrp2 Pik3cd Plcg1 Prf1 Prkcq Psmb8 Ptprc Ptprv Rassf5 Rhoh Ripk3 Sash3 Satb1 Sh2d1a Sh3kbp1 Slamf7 Slc2a3 Spn Stap1 Stat1 Stk4 Tcf7 Themis Thy1 Tnfrsf18 Trat1 Ubash3a Vav1 Zap70	Apoptotic (GO:0006915), Adhesion (GO:0022610), Regulation (GO:0065007), Cellular component organization (GO:0071840), Cellular (GO:0009987), Developmental (GO:0032502), Immune system (GO:0002376), Localization (GO:0051179), Metabolic (GO:0008152), Multicellular organismal (GO:0032501), Response to stimulus (GO:0050896), Reproduction (GO:0000003)
	miR-30e (↓)	5830411N06Rik Amigo2 Bcl11b Card11 Ccr7 Cd247 Cd27 Cd3d Cd5 Cd8a Coro1a Ets1 Il16 Itgal Jarid2 Lat Lck Nlrc3 Pglyrp2 Prkcq Ptprc Rhoh Satb1 Sh2d1a Spn Stk4 Tcf7 Trat1 Vav1	Apoptotic (GO:0006915), Adhesion (GO:0022610), Regulation (GO:0065007), Cellular component organization (GO:0071840), Cellular (GO:0009987), Developmental (GO:0032502), Immune system (GO:0002376), Localization (GO:0051179), Metabolic (GO:0008152), Multicellular organismal (GO:0032501), Response to stimulus (GO:0050896), Reproduction (GO:0000003)
	miR-103 (↓)	5830411N06Rik Amigo2 Bcl11b Btla Card11 Ccr7 Cd1d2 Cd2 Cd247 Cd27 Cd28 Cd3d Cd3e Cd4 Cd5 Cd8a Cnr2 Coro1a CskD1 Dock2 Elmo1 Ets1 Gzma Hsh2d Il16 Il27ra Il2rg Il7r Irf1 Itga lJarid2 Klhl6 Lat Lax1 Lck Madd Mgea5 Nlrc3 Pdcd1 Pglyrp2 Pik3cd Prf1 Prkcq Psmb8 Ptprc Ptprv Rassf5 Rhoh Ripk3 Sash3 Satb1 Sh2d1a Sh3kbp1 Slc2a3 Spn Stat1 Stk4 Tcf7 Themis Tnfrsf18 Trat1 Ubash3a Vav1 Zap70	Apoptotic (GO:0006915), Adhesion (GO:0022610), Regulation (GO:0065007), Cellular component organization (GO:0071840), Cellular (GO:0009987), Developmental (GO:0032502), Immune system (GO:0002376), Localization (GO:0051179), Metabolic (GO:0008152), Multicellular organismal (GO:0032501), Response to stimulus (GO:0050896), Reproduction (GO:0000003)
	miR-125a-3p (↑)	5830411N06Rik, Bcl11b, Cd247, Cd8a, Prkcq, Tcf7	Apoptotic (GO:0006915), Adhesion (GO:0022610), Regulation (GO:0065007), Cellular component organization (GO:0071840), Cellular (GO:0009987), Developmental (GO:0032502), Immune system (GO:0002376), Localization (GO:0051179), Metabolic (GO:0008152), Multicellular organismal (GO:0032501), Response to stimulus (GO:0050896), Reproduction (GO:0000003)
	miR-125b-5p (↑)	5830411N06Rik Bcl11b Cd247 Cd8a Lck Prkcq Tcf7Trat1	Apoptotic (GO:0006915), Adhesion (GO:0022610), Regulation (GO:0065007), Cellular component organization (GO:0071840), Cellular (GO:0009987), Developmental (GO:0032502), Immune system (GO:0002376), Localization (GO:0051179), Metabolic (GO:0008152), Multicellular organismal (GO:0032501), Response to stimulus (GO:0050896), Reproduction (GO:0000003)
	miR-126-3p (↑)	5830411N06Rik Bcl11b Cd247 Cd8a Lck Prkcq Tcf7	Apoptotic (GO:0006915), Adhesion (GO:0022610), Regulation (GO:0065007), Cellular component organization (GO:0071840), Cellular (GO:0009987), Developmental (GO:0032502), Immune system (GO:0002376), Localization (GO:0051179), Metabolic (GO:0008152), Multicellular organismal (GO:0032501), Response to stimulus (GO:0050896), Reproduction (GO:0000003)
	miR-139-3p (↑)	Cd247	Biological regulation (GO: 0065007)
	miR-141 (↑)	Cd247	Biological regulation (GO: 0065007)
	miR-188-5p (↑)	5830411N06Rik Bcl11b Cd247 Cd8a Tcf7	Apoptotic (GO:0006915), Adhesion (GO:0022610), Regulation (GO:0065007), Cellular (GO:0009987), Developmental (GO:0032502), Immune system (GO:0002376), Localization (GO:0051179), Metabolic (GO:0008152), Multicellular organismal (GO:0032501), Response to stimulus (GO:0050896), Reproduction (GO:0000003)
	miR-200a (↑)	Cd247	Biological regulation (GO: 0065007)
	miR-200b (↑)	Cd247	Biological regulation (GO: 0065007)
	miR-200c	Cd247	Biological regulation (GO: 0065007)
	miR-202-3p (↑)	Ccr7 CD247	Biological regulation (GO: 0065007), Cellular (GO:0009987), Immune system (GO:0002376), Multicellular organismal (GO:0032501), Response to stimulus (GO:0050896)
	miR-370 (↑)	5830411N06Rik Amigo2 Bcl11b Btla Card11 Ccr7 Cd247 Cd27 Cd3d Cd5 Cd8a Coro1a Ets1 Il16 Il2rg Il7r Itgal Jarid2 Klhl6 Lat Lck Madd Nlrc3 Pglyrp2 Prf1 Prkcq Ptprc Rhoh Sash3 Satb1 Sh2d1a Spn Stk4 Tcf7 Trat1 Vav1	Apoptotic (GO:0006915), Adhesion (GO:0022610), Regulation (GO:0065007), Cellular component organization (GO:0071840), Cellular (GO:0009987), Developmental (GO:0032502), Immune system (GO:0002376), Localization (GO:0051179), Metabolic (GO:0008152), Multicellular organismal (GO:0032501), Response to stimulus (GO:0050896), Reproduction (GO:0000003)
	miR-375 (↑)	Cd247	Biological regulation (GO: 0065007)
	miR-483 (↑)	Cd247	Biological regulation (GO: 0065007)
	miR-680 (↑)	5830411N06Rik Bcl11b Cd247 Cd8a Lck Prkcq Tcf7 Trat1	Apoptotic (GO:0006915), Adhesion (GO:0022610), Regulation (GO:0065007), Cellular (GO:0009987), Developmental (GO:0032502), Immune system (GO:0002376), Localization (GO:0051179), Metabolic (GO:0008152), Multicellular organismal (GO:0032501), Response to stimulus (GO:0050896), Reproduction (GO:0000003)

#### Microarray mRNA expression profiles (transcriptome)

From the whole set of sequences that were tested in the microarray, we initially identified a set of 33,459 mRNAs that were differentially expressed [p < 0.05, false discovery rate (FDR) = 0.05 and fold change ≥ 2.0] when comparing thymocytes from 1 mo-old and CD3^+^ peripheral T lymphocytes from 1mo-old or 7 mo-old pre-diabetic mice with the corresponding cells from ≥ 7 mo-old diabetic mice or PILs from pre-diabetic 1 mo-old NOD mice. From this set, we subsequently selected 2,070 mRNAs encoding proteins participating in biological processes associated with apoptosis, cell adhesion, cellular regulation, cellular component organization, cellular processes, development and the immune system.

**Fig 2:**
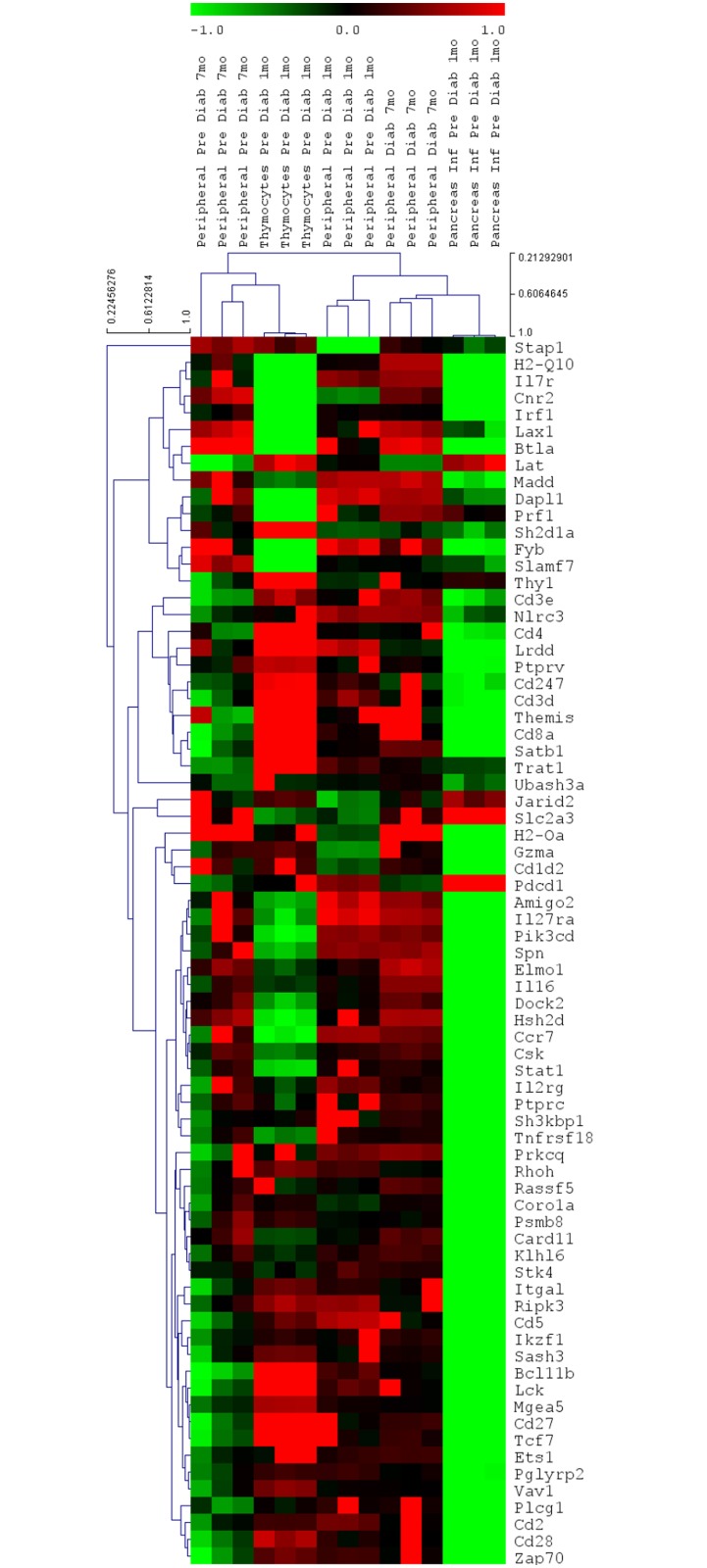
Transcriptome (mRNA) expression profiles. The samples analyzed were as follow: Thymocytes (n = 3) or pancreas-infiltrating lymphocytes (PILs) derived from pre-diabetic (1 mo) (n = 3) or peripheral CD3^+^ T lymphocytes derived from pre-diabetic [1 mo (n = 2) or 7 mo (n = 3)] NOD mice. These cell types featured unique transcriptome expression signature. The dendrograms and heat map were obtained using cluster and tree-view algorithms through the Agilent GeneSpring platform. Heat map legend: red = up-regulation, green = down-regulation, black = unmodulated (Pearson correlation metrics).

The dendrogram and heat map depicted in Fig 2 show that these cell types feature unique expression signatures encompassing 73 mRNAs. In this work, we used a FACS sorted PIL preparation from which we focused those genes involved in the molecular/biological processes considered relevant for autoimmune reactivity for further analysis. The mRNAs of each cell type were then reanalyzed using the GenMir++ algorithm.

### Reconstruction of miRNA-mRNA interaction networks

**Fig 3:**
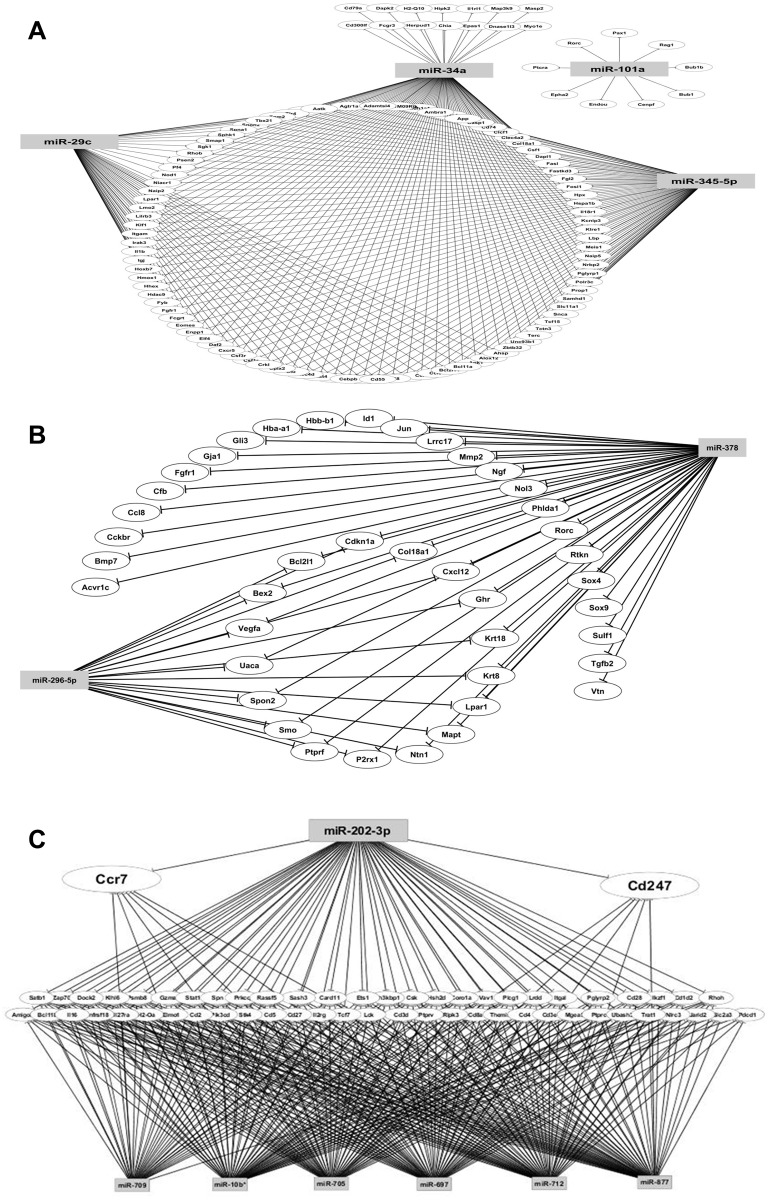
Posttranscriptional miRNA-mRNA interactions observed in thymocytes (A), peripheral CD3+ T lymphocytes (B) or pancreas-infiltrating lymphocytes (C) isolated from pre-diabetic or diabetic, non-obese diabetic (NOD) mice. The differentially expressed (up- or down-regulated) miRNAs and differentially expressed mRNAs were reanalyzed using the GenMir++ algorithm to reconstruct the interaction networks.

The 13 selected differentially expressed (up- or down-regulated) miRNAs and 2,070 selected differentially expressed (up- or down-regulated) mRNAs were reanalyzed using the GenMir++ algorithm to reconstruct the miRNA-mRNA interaction predictions. The predicted miRNA-mRNA interaction networks are depicted according to cell type: thymocytes (Fig 3A), CD3^+^ peripheral T lymphocytes (Fig 3B) and PILs (Fig 3C).

In a comparative analysis involving thymocytes derived from 4 week-old (1 mo) pre-diabetic, T CD3^+^ peripheral lymphocytes derived from pre-diabetic (1 mo or 7 mo) and diabetic (≥ 7 mo), PILs derived from pre-diabetic (1 mo) NOD mice we observed that thymocytes exhibited four modulated miRNAs, of which two were down-regulated (miR-101a and miR-29c) and two were up-regulated (miR-345-5p and miR-34a). CD3^+^ peripheral T lymphocytes exhibited two modulated miRNAs, miR-296-5p (down-regulated) and miR-378 (up-regulated), and PILs exhibited seven upregulated miRNAs (miR 202-3p, miR 709, miR 10b*, miR 705, miR 697, miR 712 and miR 877). Both types of modulated miRNAs (down- or up-regulated) interacted with mRNA targets largely involved in the following gene-ontology (GO) processes: the immune system, apoptosis, cell adhesion, cell regulation, cellular component organization, cellular processes, development, localization, metabolism, multicellular organismal, reproduction and response to stimulus (Table 1).

We determined that thymocytes featured the following mRNAs targets, among others, with their molecular/biological processes in parentheses: RAG-1 (VDJ recombination); Ptcra (pre-T-cell antigen receptor alpha); Ccl3, Ccl6 and Pf4 (chemokine activity); Fastkd3 (cell cycle); Lmo2 (immune system and apoptosis, among others); and Clec4d, Cd36 and Cd55 (cell adhesion). The CD3^+^ peripheral T lymphocytes featured, among others, Cdkn1a (cell cycle), Col18a1 (cell adhesion) and Vegfa (cell communication).

The PILs featured, among others, Cd27 antigen (negative regulation of apoptosis, among several others); ItgaL and Prf1 (cell adhesion); Cd28 (cell-cell signaling); and Cd3e (cell-cell communication and immune system).

We decided to further study the participation of Ccr7 and Cd247 (Cd3 zeta chain) in PILs that were simultaneously regulated by several miRNAs, including up-regulated miR-202-3p, due to the involvement of these mRNAs in the control of cellular autoimmune reactivity. Because of their relevance to this study and their possible involvement in the control of auto-reactivity, these miRNA-mRNA interactions were selected for further validation.

### miR-202-3p or Cd247 mRNA expression levels as evaluated by qRT-PCR

**Fig 4:**
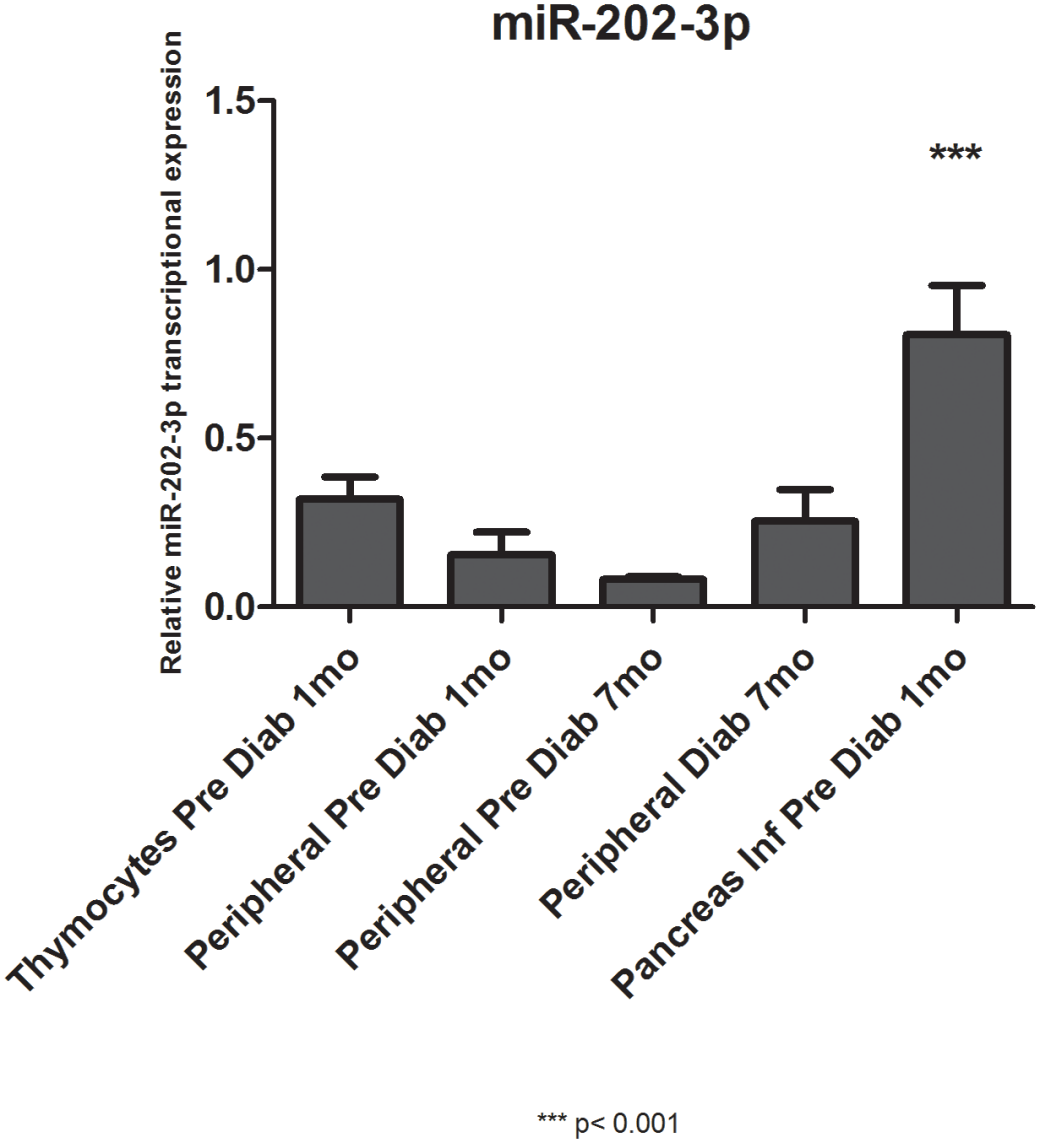
Relative transcriptional levels of miR-202-3p as evaluated by quantitative reverse-transcription PCR (qRT-PCR). Taqman qRT-PCR was used to evaluate the levels of miR-202-3p while thymocytes evolve into peripheral T CD3^+^ lymphocytes and PILs. PD = pre-diabetic, mo = months.

**Fig 5:**
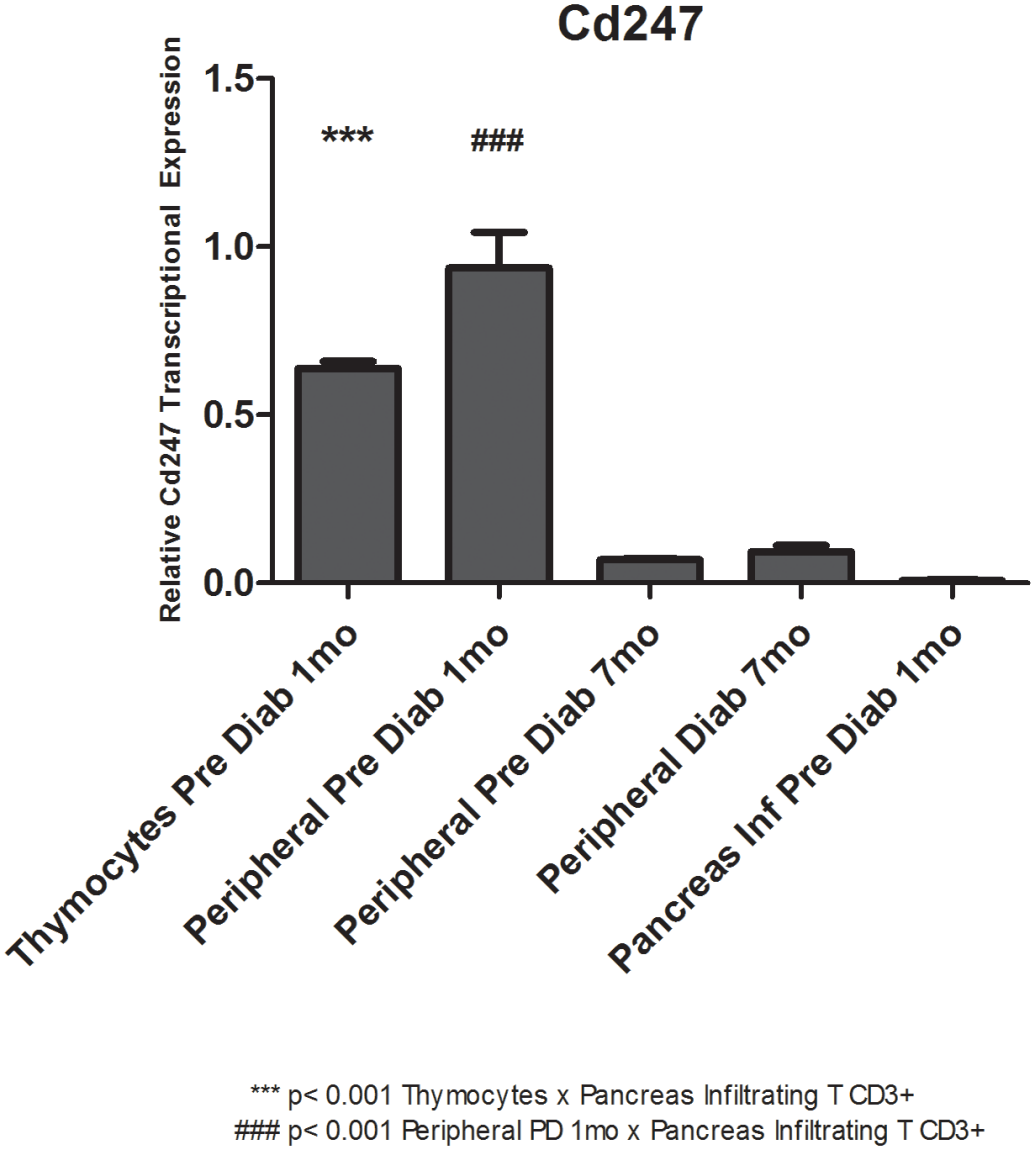
Relative transcriptional levels of Cd247 mRNA as evaluated by quantitative reverse-transcription PCR (qRT-PCR). Taqman qRT-PCR was used to evaluate the levels of Cd247 mRNA while thymocytes evolve into peripheral T CD3^+^ lymphocytes and PILs. PD = pre-diabetic, mo = months.

We observe that while thymocytes develop into peripheral T CD3^+^ lymphocytes and PILs, the expression levels of miR-202-3p and Cd247 mRNA are opposite. While miR-202-3p levels are relatively lower in thymocytes and in peripheral T CD3^+^ lymphocytes, the CD247 mRNA levels are higher. The opposite occurs when we compare their expression in PILs. In these cells, miR-202-3p levels are high while Cd247 mRNA is low (Figs 4 and 5).

### Validation of miRNA-mRNA interactions

#### Minimal free energy (RNA-Hybrid algorithm)

The consequential pairing of the *M*. *musculus* Ccr7 mRNA (acc NM_001838) target region and the miR-202-3p miRNA was predicted to occur at nucleotide positions 166–172 of its 760-bp 3´ UTR (www.targetscan.org). This miR-202-30p Cd247 (*M*. *musculus* Cd247 mRNA, acc. NM_ 000734 or NM_198053) interaction has not been previously reported. To validate our findings, we used the RNA-Hybrid bioinformatics tool, which calculates the most favorable hybridization between a given miRNA and the predicted 3´ UTR portion of its mRNA target by calculating the minimum free energy (MFE).

**Fig 6:**
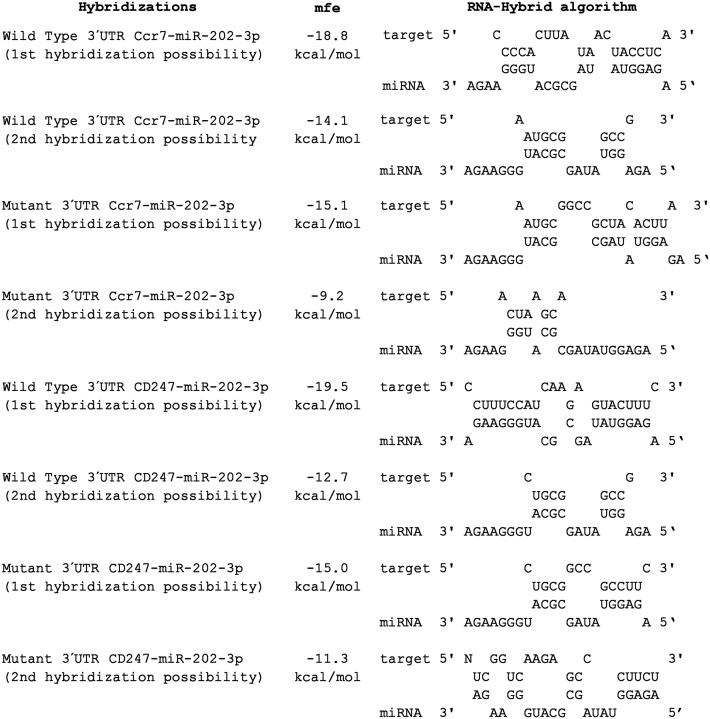
Hybridization likelihoods between the wild type and mutant 3´UTRs of the Ccr7 and Cd247 mRNA targets and miR-202-3p. The miRNA:mRNA hybrid structures and their respective minimum free energies were calculated using the RNA hybrid algorithm. Mfe = minimum free energy.

The respective miRNA-mRNA annealing and MFEs are presented in Fig 6. Artificially introduced mutations changed the MFE and likelihood of miRNA-mRNA hybridization.

#### Luciferase reporter gene assay (LRGA)

**Fig 7:**
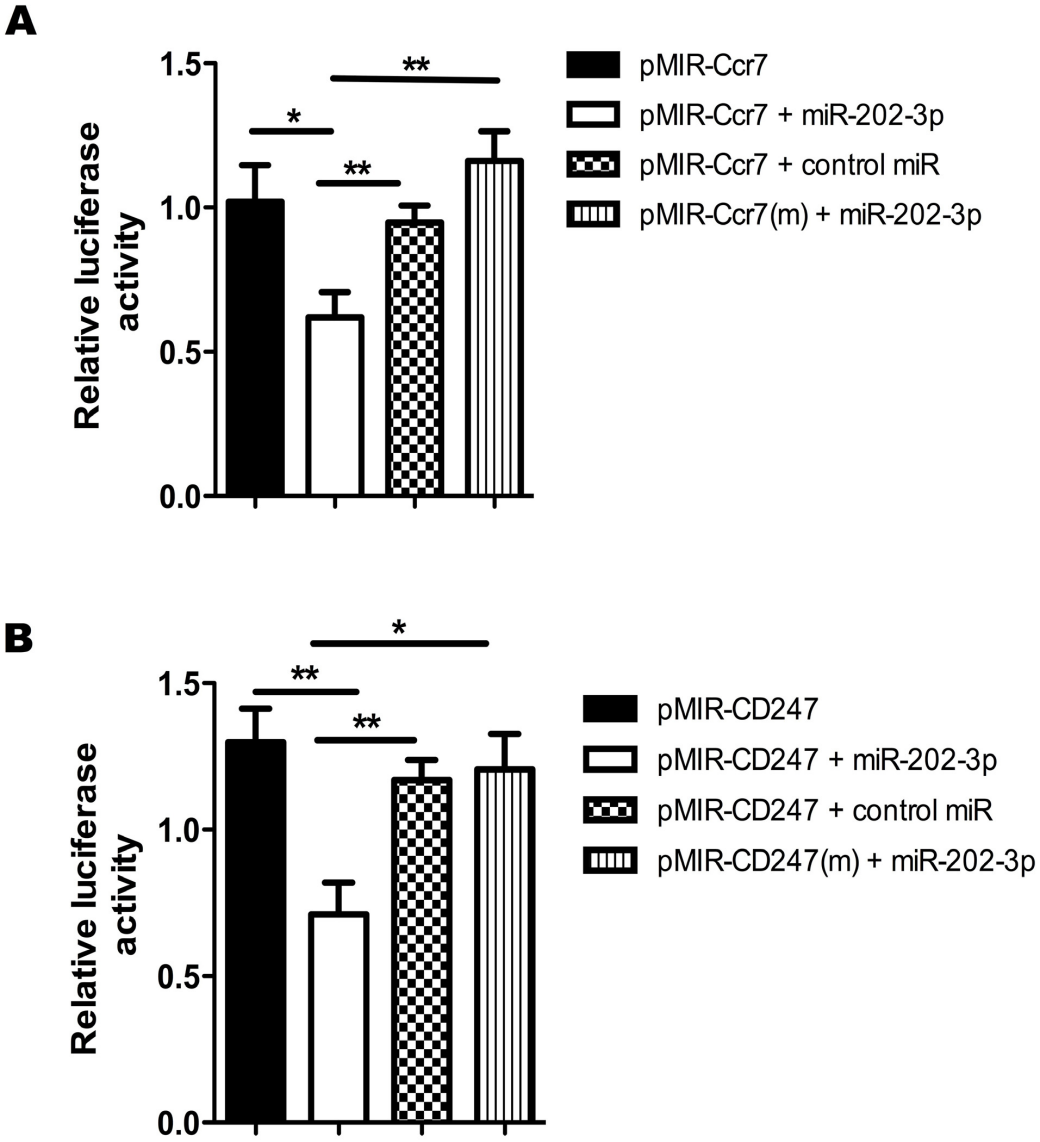
Luciferase reporter gene assay (LRGA). Posttranscriptional interactions between miR-202-3p-Ccr7 (A) or miR-202-3p-Cd247 (B) were assessed by LRA.pMIR-Ccr7 or pMIR-Ccr7(m) and pMIR-Cd247 or pMIR-Cd247(m) 3´ UTR luciferase plasmid constructs were transfected into human HEK-293T cells. Cotransfections with miR-202-3p or control (scrambled) miRNA were also performed to show the specificity of the miRNA, and co-transfections with the mutant (m) plasmid constructs were performed to show specificity of the 3´ UTR for the interactions. The data are presented as the means and standard error of mean (SEM). The differences were evaluated by one-way ANOVA followed by Bonferroni´s test, and p < 0.05 was considered statistically significant when the wild-type 3´ UTR pMIR was compared to the mutant (m) 3´ UTR sequence in the presence of the respective miRNA mimic.

The LRGA was used to confirm the occurrence of the miR-202-3p- Ccr7 or miR-202-3p-Cd247 3´ UTR interactions within the cellular milieu. We confirmed that miR-202-3p interacts with Ccr7 or Cd247 by its hybridization to their respective 3´ UTR sequences, which contain the predicted binding sites for this miRNA. Irrelevant miRNA sequences or artificially introduced point mutations into (base substitutions) or base deletions from the original 3´ UTRs significantly abolished the miRNA interaction, thus demonstrating the necessity of specificity and the exactness of base pairing for hybridization within the nucleotides that were selected for mutation (Fig 7).

## Discussion

Type 1 diabetes mellitus (T1D) is an autoimmune metabolic disease with a strong genetic influence and association with environmental components. T1D begins with inflammatory insulitis, i.e., autoreactivity mediated mainly by CD4+ and/or CD8^+^ T lymphocytes that are directed toward insulin-producing pancreatic beta cells, in prone individuals or non-obese diabetic (NOD) mice [15,23,50–52]. Efforts are being made to better characterize the pancreatic autoantigens that are involved in insulitis as well as autoreactive immune cells [53–55].

In a previous study [15], we demonstrated the transcriptional modulation of immune reactivity genes during the development of T1D as thymocytes mature into peripheral CD3^+^ T lymphocytes in NOD mice, whereby genes involved in the negative selection of thymocytes, T-cell maturation, differentiation and autoreactivity are sequentially expressed.

However, two important gaps remain: the assessment of gene expression of the T lymphocytes that invade the pancreas (pancreas-infiltrating lymphocytes or PILs) and the evaluation of post-transcriptional control involving miRNAs.

In the present study, we sought to fill in these gaps by following a more comprehensive approach. Our hypothesis was twofold: first, we hypothesized that the modulation (up- or down-regulation) of miRNAs or mRNAs would be sufficient to hierarchize the different cell types (thymocytes, CD3^+^ peripheral T lymphocytes and PILs) as well as the different stages of NOD mice (pre- or diabetic); second, we examined the interaction between modulated miRNAs and mRNAs from these cells during the evolution of T1D in these mice.

Our intention was to evaluate the expression signatures of these cells by focusing on known miRNA or mRNA sequences. The microarray method and bioinformatics pipeline that were used for this data analysis were adequate for this purpose. As proof of the suitability of these methods, the dendrograms and heat maps depicted in Figs 1 and 2 show that the different cell samples that were collected at different phases of NOD mouse development featured distinctive miRNA or mRNA expression signatures.

Moreover, to reconstruct the miRNA-mRNA interaction networks, the data were analyzed using the GenMir++ algorithm [46,47], which is based on Bayesian statistics for the analysis of large data sets originating from microarray hybridizations.

The results revealed that thymocytes, peripheral CD3^+^ T lymphocytes and PILs featured a large set of miRNAs as well as mRNAs that were differentially modulated (up- or down-regulated) (Table 1). As a result, these cells, as well as the different phases of diabetic evolution in the mice (pre- or diabetic), were hierarchically clustered according to their respective miRNA or mRNA expression signatures. These data confirmed the first part of the original hypothesis.

Moreover, the demonstration of miRNA modulation corresponded to a unique aspect of this work and indicated that these cells undergo posttranscriptional control. By using Bayesian statistics through the GenMir++ algorithm, we were able to reconstruct miRNA-mRNA interaction networks and predict mRNA targets in an unbiased way, thus demonstrating that differentially expressed miRNAs interact with a large set of mRNA targets in each T lymphocyte phase that was assayed (Fig 3A–3C). This result confirmed the second part of the hypothesis.

As expected, the miRNAs that were selected for the reconstruction of the networks simultaneously interacted with several mRNA targets. For example, miR-101a, which was down-regulated in thymocytes, interacted with the following mRNA targets: Bub1, Bub1b, Cenpf, Endou, Rorc, Pax1, Epha2, Rag1 and Ptcra.

In addition, a given mRNA was regulated by several miRNAs. For example, in thymocytes, the Ccl3 mRNA simultaneously interacted with the following miRNAs: miR-29c (down-regulated), miR-345-5p and miR-34a (up-regulated).

Moreover, we observed that, independent of the expression profile (up- or down-regulation), miRNAs interacted with mRNA targets. The modulation of miRNAs was comparative among the cell types i.e. thymocytes, CD3^+^ peripheral T lymphocytes and PILs.

Regardless of their expression profile (up- or down-regulation), modulation of the miRNAs predicted the miRNA-mRNA interactions. This presumption was reinforced when we observed the opposite levels of miR-202-3p and Cd247 mRNA target during the development of thymocytes into peripheral T CD3^+^ lymphocytes and PILs (Figs 4 and 5).

The interactions of greater interest for this study were validated at two levels. In the first, we evaluated the minimum free energy (MFE) of the annealing between the miRNA and 3´ UTR seed region of its mRNA target (Fig 6). The second level of validation involved the luciferase reporter assay, which confirmed that the miRNA-mRNA interaction could occur in the cell milieu (Fig 7).

As a whole, as they develop into CD3^+^ peripheral T cells and then into PILs, thymocytes exhibit miRNA interactions involving mRNA targets related to the immune system, apoptosis, cell adhesion, cellular regulation, cellular component organization, cellular processes, development, positive or negative thymic selection (although this miRNA regulation is absent in thymocytes) and immunological synapse, among others.

The T lymphocytes that were analyzed at different stages during the evolution of T1D in NOD mice underwent complex posttranscriptional control.

In our view, PILs are a cell type that should be better studied with respect to their posttranscriptional control of gene expression. Although efforts are being made to better understand the role of these cells [56], to date, little is known about their gene expression control.

In a more focused examination of PILs, we observed that miR-202-3p regulates the Ccr7 and Cd247 (CD3 zeta chain) mRNAs. This result is intriguing because Ccr7 is involved in the control of tolerance, and a previous study [57] demonstrated that mice lacking this chemokine receptor generated autoreactive T cells.

Moreover, Cd247 (CD3 zeta chain) was identified as a novel diabetes susceptibility gene whose function positively influenced the onset of autoimmune diabetes in mice when the gene was disturbed during TCR signaling [58].

Using the strategy of large-scale miRNA-mRNA interaction screening, in this study, we present evidence that these two mRNAs are under posttranscriptional control in PILs of NOD mice.

In our view, these results open new perspectives for further studies involving the use of miRNA antagonists (anti-Mirs) that are directed toward miR- 202-3p and other interacting miRNAs, which could be further assayed in the control of insulitis *in vivo* in NOD mice and/or *in vitro* making use of the transwell assay to evaluate the migration of NOD mice-derived T lymphocytes toward pancreatic autoantigens.

## Supporting Information

S1 Fig Graphics generated by FACS Aria III device during the separation of pancreas infiltrating CD3+ T lymphocytes (PILs). The PILs were separated with a purity of 70.8%.
(PDF)
