# Supplementary figures and images for: Comprehensive Survey of miRNA-mRNA Interactions Reveals That Ccr7 and Cd247 (CD3 zeta) are Posttranscriptionally Controlled in Pancreas Infiltrating T Lymphocytes of Non-Obese Diabetic (NOD) Mice
Source: PLoS One. 2015 Nov 25;10(11):e0142688. doi: 10.1371/journal.pone.0142688 (PMC4659659; doi:10.1371/journal.pone.0142688)

# FACSDiva Version 6.1.3

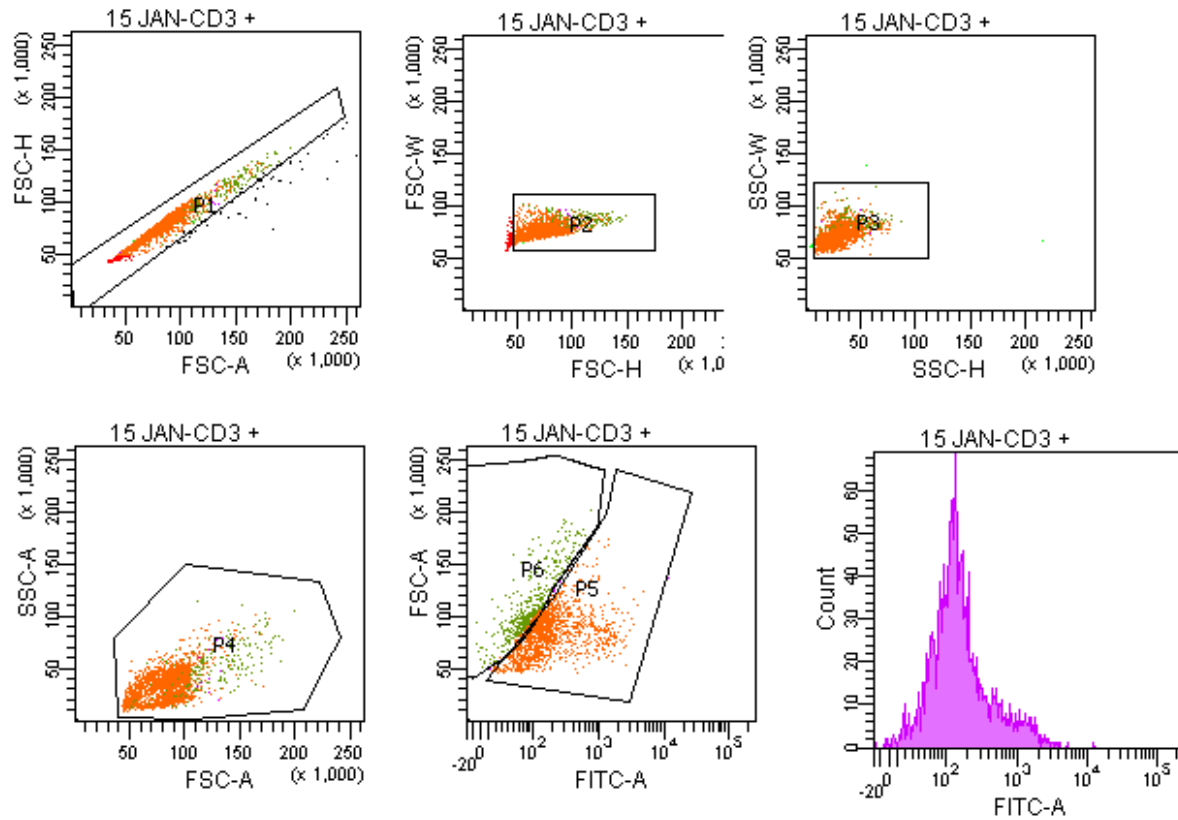

Tube: CD3 +

| Population | #Events | %Parent | %Total |
|------------|---------|---------|--------|
| All Events | 2,168   | ####    | 100.0  |
| P1         | 2,105   | 97.1    | 97.1   |
| P2         | 1,971   | 93.6    | 90.9   |
| P3         | 1,964   | 99.6    | 90.6   |
| P4         | 1,964   | 100.0   | 90.6   |
| P6         | 509     | 25.9    | 23.5   |
| P5         | 1,535   | 78.2    | 70.8   |

Supplement: S1 Fig — The PILs were separated with a purity of 70.8%. (PDF) [file pone.0142688.s001.pdf]
